# When universities advocate GAI but practice falls short: student appraisals and creative process engagement in art education

**DOI:** 10.3389/fpsyg.2026.1955192

**Published:** 2026-09-25

**Authors:** Yuantao Chen

**Affiliations:** Advanced School of Art and Humanities, China Academy of Art, Hangzhou, China

**Keywords:** cognitive appraisal, creative engagement, generative AI, higher education, institutional inconsistency, pathways thinking

## Abstract

**Introduction:**

Universities are increasingly promoting generative artificial intelligence (GAI) in art education; however, institutional advocacy is not always matched by consistent implementation. Drawing on the transactional theory of stress and coping, this study examines how GAI advocacy–practice inconsistency relates to art students’ creative process engagement through challenge and hindrance appraisals and whether pathways thinking shapes these processes.

**Methods:**

A two-study design was employed. Study 1 employed a classroom-based between-subjects scenario experiment involving 160 art students to compare challenge and hindrance appraisals under high- and low-inconsistency conditions. Study 2 surveyed 425 students with recent experience of GAI-assisted art learning and tested the proposed direct, indirect, moderating, and conditional indirect relationships using PLS-SEM.

**Results:**

In Study 1, participants in the high-inconsistency scenario reported stronger challenge and hindrance appraisals than those in the low-inconsistency scenario, although the manipulation captured a broader experience of inconsistent implementation rather than an isolated word–deed inconsistency effect. In Study 2, inconsistency showed a significant negative indirect association with creative process engagement through hindrance appraisal and a significant positive indirect association through challenge appraisal. Pathways thinking strengthened the association between inconsistency and challenge appraisal and the corresponding positive conditional indirect association, but did not significantly weaken either the hindrance appraisal relationship or the negative indirect pathway.

**Discussion:**

Institutional inconsistency does not produce a uniform student response; instead, it can be interpreted simultaneously as an obstacle and as a manageable developmental demand, with opposing implications for creative engagement. The findings extend research on institutional word–deed alignment to GAI-supported art education and identify pathways thinking as a resource that facilitates challenge-oriented interpretation without eliminating awareness of genuine institutional constraints. Universities should align GAI commitments with teaching, assessment, and support practices while helping students develop alternative routes for coping with implementation uncertainty.

## 1 Introduction

Generative artificial intelligence (GAI) is expanding the possible boundaries of artistic creation. Generative tools for images, text, music, video, and multimodal content are reducing the time and technical costs of content production. Beyond improving creative efficiency, these tools can also support creative exploration, idea development, and iterative refinement, and are gradually being integrated into university art courses and students’ creative practices (Hwang and Lee, 2025; Wu et al., 2026). Given these potential benefits, an increasing number of universities and art schools have begun to advocate the responsible use of GAI and promote its integration into art education through instructional planning, usage guidelines, and public commitments (Jin et al., 2025; Wang et al., 2024). However, such advocacy does not always translate into corresponding educational practices. Actual implementation, instructor requirements, assessment approaches, and the handling of related issues may diverge from institutions’ public statements and prior commitments. Therefore, what students face is not merely whether instructional support is sufficient, but whether they perceive consistency between what institutions advocate and what they actually do. This study refers to this perceived discrepancy between institutional GAI-related commitments and subsequent practices as GAI advocacy–practice inconsistency.

Existing research on GAI in higher education has examined its educational applications from multiple perspectives. From a learner perspective, studies have mainly focused on GAI usage intention, perceived benefits, and potential risks (Chan and Hu, 2023; Shen et al., 2025; Zhu et al., 2025a). From an institutional governance perspective, scholars have examined university GAI policies, academic integrity guidelines, and instructional support arrangements (Jin et al., 2025; McDonald et al., 2025; Wang et al., 2024). In addition, some studies have further analyzed the effects of GAI on learning performance, creativity, and human–AI co-creation experiences (Habib et al., 2024; Heigl, 2026; Zhu et al., 2025b). Although these studies have, respectively, examined individual responses, institutional responses, and learning outcomes, insufficient attention has been paid to the consistency between universities’ public advocacy and their actual practices. Therefore, whether institutions are consistent in what they advocate and what they actually do in GAI-based art education, and how such inconsistency affects students, remains to be systematically examined.

According to the transactional theory of stress and coping (TTSC), individuals’ responses to environmental demands are shaped by their appraisals of how those demands affect valued goals, growth prospects, and available coping resources (Lazarus and Folkman, 1984; Biggs et al., 2017). From this perspective, institutional GAI advocacy–practice inconsistency may be appraised as a hindrance because of conflicting rules, unpredictable assessment, and unfulfilled commitments, but it may also be interpreted by some students as a challenge that requires them to adjust their strategies, seek resources, and explore alternative solutions (Webster et al., 2011; Podsakoff et al., 2023). Prior research has shown that the same environmental demand may lead to different outcomes depending on whether it is appraised as a challenge or a hindrance (LePine et al., 2016; Kunzelmann et al., 2025). This appraisal process may also be shaped by pathways thinking, which refers to individuals’ tendency to generate multiple viable routes to goal attainment and to develop alternatives when existing routes are blocked (Snyder et al., 1991). Recent research further suggests that hope can enhance individuals’ sense of control and adaptability under stressful and uncertain conditions (Gallagher et al., 2021). Students with stronger pathways thinking may be more likely to identify multiple courses of action and, in turn, appraise the same institutional GAI advocacy–practice inconsistency in different ways.

These differing appraisals may, in turn, shape whether students become genuinely engaged in GAI-assisted artistic creation. Creative process engagement emphasizes individuals’ sustained involvement in cognitive activities such as problem identification, information searching and encoding, and idea generation during the creative process (Zhang and Bartol, 2010). In the GAI context, obtaining technologically generated content is not equivalent to creative engagement; meaningful art learning is more likely to occur when students critically interpret the task, evaluate and select relevant information, consider alternative possibilities, and iteratively refine the generated outputs (Hwang and Lee, 2025; Georgieva and Georgiev, 2025). Challenge appraisal may lead students to view environmental demands as opportunities for growth and goal attainment, thereby sustaining their engagement in these complex creative activities. By contrast, hindrance appraisal may lead students to believe that their efforts are unlikely to produce effective outcomes, thereby weakening their willingness to continue exploring and processing information (Podsakoff et al., 2023). Although existing research has extensively discussed the effects of GAI on creativity and learning outcomes (Habib et al., 2024; Heigl, 2026; Zhu et al., 2025b), systematic analysis remains limited regarding how students’ perceived GAI advocacy–practice inconsistency in their institutional environment relates to engagement in specific creative processes through cognitive appraisals.

Accordingly, this study examines how GAI advocacy—practice inconsistency in institutional contexts is associated with art students’ challenge and hindrance appraisals of an ongoing GAI-supported learning environment, whether these associations vary with pathways thinking, and how the two appraisals are further associated with students’ creative process engagement. By focusing on the relationship between what institutions advocate and what they actually do, this study extends existing research on GAI in higher education beyond policy formulation, technology adoption, and support conditions to consider institutional word–deed alignment. Furthermore, drawing on the TTSC, this study examines students’ perceptions of GAI advocacy–practice inconsistency in their institutional environment and how these perceptions are associated with their cognitive appraisals, while considering pathways thinking as a boundary condition that may shape students’ interpretations of the same institutional environment. Finally, by linking challenge and hindrance appraisals to creative process engagement, this study extends the discussion of GAI-related educational outcomes beyond whether students use the technology and its general outcomes to whether they genuinely engage in core creative processes.

## 2 Review and hypothesis development

### 2.1 A cognitive appraisal perspective on GAI advocacy–practice inconsistency

The TTSC holds that environmental conditions produce psychological and behavioral consequences only when individuals appraise them as relevant to important goals (Lazarus and Folkman, 1984; Biggs et al., 2017). Challenge appraisal refers to an individual’s judgment that a demand, although requiring effort, contains the potential for growth, mastery, or goal attainment, whereas hindrance appraisal reflects the judgment that the demand will constrain action and impede goal progress. These two appraisals are not inherent properties of environmental demands; rather, they are relational judgments formed by individuals in light of goal importance, controllability, and available coping resources. Accordingly, the same environmental demand may simultaneously carry both challenge and hindrance meanings, making cognitive appraisal a key mechanism linking external conditions to individual responses (Webster et al., 2011; LePine et al., 2016; Podsakoff et al., 2023). This view also aligns with the central proposition of the newer challenge–hindrance framework: researchers should not infer the consequences of stressors solely from their objective categories, but should directly examine the meanings individuals assign to those demands.

GAI advocacy–practice inconsistency is precisely such an institutional environmental demand that requires interpretation by students. Research on organizational word–deed alignment shows that members attend to the degree of correspondence between an organization’s public statements and its actual actions, and use this correspondence to judge whether its commitments are credible and whether subsequent actions are predictable (Simons, 2002; Simons et al., 2022; Kılıçoğlu et al., 2019). When words and practices diverge, the action cues provided by the organization become ambiguous, prompting students to reassess whether their goals can be achieved and whether they possess sufficient resources to cope with the situation (Webster et al., 2011; Podsakoff et al., 2023). For art students, such inconsistency may signify institutional constraints, uncertainty in implementation, and obstruction of learning goals, but it may also indicate a need to proactively adjust their approaches, seek alternative resources, and develop new coping pathways. Importantly, GAI advocacy–practice inconsistency should not be interpreted as a stressor with a single predetermined meaning. Rather, it represents a discrepancy that may generate multiple goal-relevant interpretations. On the one hand, inconsistent implementation may create uncertainty regarding acceptable behaviors, available resources, and expected outcomes, thereby increasing hindrance appraisal. On the other hand, when students perceive possibilities for adaptation and problem solving, the same situation may also stimulate challenge appraisal. Thus, challenge and hindrance appraisals represent distinct evaluations of the same environmental condition rather than mutually exclusive categories (Webster et al., 2011; Searle and Auton, 2015; Podsakoff et al., 2023). Accordingly, this study does not treat GAI advocacy–practice inconsistency as a stressor with a fixed nature. Instead, it conceptualizes it as an environmental condition that triggers students’ challenge and hindrance appraisals, and uses these two appraisals to explain why it may subsequently be associated with different levels of creative process engagement.

### 2.2 The hindrance pathway from GAI advocacy–practice inconsistency to creative process engagement

When an institution publicly advocates the integration of GAI into art learning but fails to implement this commitment consistently in course arrangements, instructor practices, resource support, and assessment standards, students receive conflicting institutional cues. Related research indicates that a disconnect between public claims and actual actions weakens the credibility of institutional commitments and the predictability of the environment, making it difficult for members to determine which behaviors will be supported and whether their efforts will produce the expected outcomes (Kılıçoğlu et al., 2019; Simons et al., 2022; Liao et al., 2024). According to the TTSC, such uncertainty prompts students to reassess whether environmental demands interfere with their learning and creative goals (Podsakoff et al., 2023; Stephens et al., 2024). When students must devote additional effort to identifying acceptable boundaries of use, reconciling inconsistent instructional requirements, and dealing with uncertainty regarding assessment consequences, they are more likely to perceive the environment as constraining effective action and making goal attainment more difficult. Because hindrance appraisal reflects an individual’s judgment that environmental demands impede goal progress (Webster et al., 2011; Podsakoff et al., 2023), the greater the GAI advocacy–practice inconsistency, the more likely students are to form a hindrance appraisal of the GAI-supported art-learning environment. Therefore:

*H1a*: GAI advocacy–practice inconsistency is positively associated with students’ hindrance appraisal of the GAI-supported art-learning environment.

Hindrance appraisal may constitute an explanatory pathway linking GAI advocacy—practice inconsistency with creative process engagement. When students perceive the current environment as obstructing goal attainment, they are likely to lower their expected returns from continued investment and allocate less time, attention, and cognitive effort to the situation (Inam et al., 2021; Liu et al., 2022; Wang and Li, 2024). This is particularly important for creative process engagement because problem identification, information screening, and idea generation and refinement all depend on sustained cognitive processing and repeated exploration (Zhang and Bartol, 2010; Inam et al., 2021; Liu et al., 2022). When students appraise the GAI-supported art-learning environment as a hindrance, they are more likely to reduce their in-depth analysis of creative problems, comparison of different generated outputs, and continued revision of proposed solutions. GAI advocacy–practice inconsistency may show a negative indirect relationship with students’ creative process engagement through hindrance appraisal. Thus:

*H1b*: GAI advocacy–practice inconsistency is negatively associated with students’ creative process engagement indirectly through hindrance appraisal.

### 2.3 The challenge pathway from GAI advocacy–practice inconsistency to creative process engagement

GAI advocacy–practice inconsistency is not necessarily understood only as a hindrance (Stephens et al., 2024). Because challenge appraisal depends on whether students can still identify possibilities for growth, mastery, and goal attainment within environmental demands, the same institutional gap may also be interpreted as a complex task that requires proactive coping (LePine et al., 2016; Podsakoff et al., 2023; Stephens et al., 2024). When an institution’s actual implementation does not fully realize its GAI advocacy, students may need to reinterpret the boundaries of acceptable use, seek alternative sources of support, and adjust their existing learning and creative strategies. Although these demands increase the difficulty of coping, they may also provide opportunities for students to develop independent judgment, technological adaptability, and problem-solving ability. In GAI-assisted art learning in particular, students often need to explore different tools and prompting approaches, repeatedly evaluate and revise generated outputs, and accordingly adjust their creative methods and expand their expressive options (Hwang and Lee, 2025; Georgieva and Georgiev, 2025). Therefore, when students perceive that such inconsistency can still be managed through proactive adjustment and may contribute to the development of their abilities, they may form a challenge appraisal of the GAI-supported art-learning environment. Accordingly:

*H2a*: GAI advocacy–practice inconsistency is positively associated with students’ challenge appraisal of the GAI-supported art-learning environment.

Challenge appraisal may further link this institutional condition to greater engagement in the creative process. When students perceive the current environment as difficult but still conducive to learning, growth, or goal attainment, they are more likely to invest time, attention, and cognitive effort and to continue seeking effective ways of coping (Kronenwett and Rigotti, 2022; Kunzelmann et al., 2025; Liao et al., 2026). This tendency to invest effort closely aligns with the requirements of creative process engagement because GAI-assisted artistic creation involves not only obtaining generated outputs, but also actively defining problems, searching for and screening information, comparing different outputs, and continuously refining creative solutions (Zhang and Bartol, 2010; Hwang and Lee, 2025). Students who appraise the GAI-supported art-learning environment as a challenge are more likely to transform institutional uncertainty into motivation for exploration and adjustment, thereby strengthening their in-depth analysis of creative problems, information processing, and iterative refinement of solutions. Based on this logic, GAI advocacy–practice inconsistency may indirectly promote students’ creative process engagement by strengthening challenge appraisal. Accordingly:

*H2b*: GAI advocacy–practice inconsistency is positively associated with students’ creative process engagement indirectly through challenge appraisal.

### 2.4 The moderating role of pathways thinking

Pathways thinking reflects individuals’ capacity to generate viable routes toward valued goals and to develop alternative routes when existing routes are blocked (Snyder et al., 1991; Snyder, 2002). From an appraisal perspective, whether an environmental demand is perceived as a hindrance depends partly on the extent to which it is seen as obstructing goal attainment and on the response options available to the individual (Webster et al., 2011; Podsakoff et al., 2023). When GAI advocacy–practice inconsistency creates unclear requirements, uneven support, or conflicting implementation, students with stronger pathways thinking may be better able to identify alternative ways of pursuing their learning and creative goals. The availability of such alternatives reduces reliance on any single institutional pathway, making inconsistent implementation less likely to be interpreted as completely blocking goal progress. Pathways thinking may therefore weaken the extent to which GAI advocacy–practice inconsistency translates into hindrance appraisal. However, alternative routes do not remove the institutional constraints themselves, and direct empirical evidence for this buffering effect remains limited. Related research on broader personal resources has likewise found stronger associations with challenge appraisal than with reduced hindrance appraisal (Mockałło and Widerszal-Bazyl, 2021). Thus, although the theoretical logic suggests a buffering effect, the expectation should be considered cautiously:

*H3a*: Pathways thinking is expected to weaken the positive relationship between GAI advocacy–practice inconsistency and hindrance appraisal, such that this relationship is weaker when pathways thinking is high.

Pathways thinking has a clearer theoretical basis for strengthening challenge appraisal. Challenge appraisal reflects the judgment that a demanding situation, although requiring effort, remains manageable and offers possibilities for growth or goal attainment (LePine et al., 2016; Podsakoff et al., 2023). Individuals with stronger pathways thinking are better able to generate alternative routes when existing routes toward their goals are obstructed (Snyder, 2002). Recent evidence also indicates that pathways thinking is positively associated with students’ adaptation to demanding educational environments (Deng et al., 2025). Accordingly, when institutional GAI implementation falls short of stated commitments, these students may be more likely to identify feasible responses, such as seeking alternative support, adjusting their use of GAI, or reorganizing their creative strategies. By preserving viable routes toward goal attainment, pathways thinking can make institutional inconsistency appear more manageable and amenable to active problem solving, thereby strengthening challenge appraisal. Thus:

*H3b*: Pathways thinking is expected to strengthen the positive relationship between GAI advocacy–practice inconsistency and challenge appraisal, such that this relationship is stronger when pathways thinking is high.

These moderating effects may further condition the indirect relationships between GAI advocacy–practice inconsistency and creative process engagement. If pathways thinking weakens the extent to which inconsistency evokes hindrance appraisal, the corresponding negative indirect relationship through hindrance appraisal should also become weaker. Because this prediction relies on the same buffering mechanism underlying H3a, for which direct empirical evidence remains limited, the corresponding conditional indirect expectation should likewise be interpreted cautiously. Conversely, if pathways thinking strengthens the extent to which inconsistency evokes challenge appraisal, the corresponding positive indirect relationship through challenge appraisal should become stronger. Therefore:

*H3c*: The negative indirect relationship between GAI advocacy–practice inconsistency and creative process engagement through hindrance appraisal is expected to be weaker when pathways thinking is high.

*H3d*: The positive indirect relationship between GAI advocacy–practice inconsistency and creative process engagement through challenge appraisal is expected to be stronger when pathways thinking is high.

In summary, we propose a dual-path model in which GAI advocacy–practice inconsistency is associated with students’ creative process engagement through hindrance and challenge appraisals. The model further incorporates pathways thinking as a proposed boundary condition that may differentially shape these two indirect relationships, as illustrated in Figure 1.

**Figure 1:**
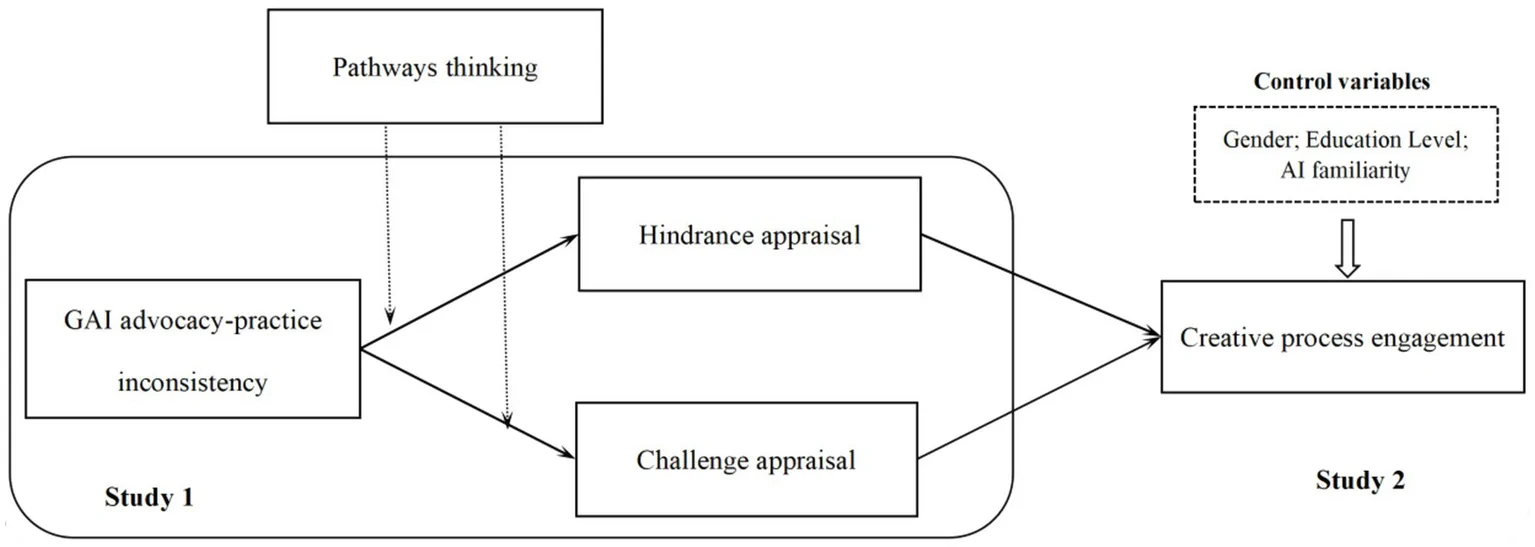
The research model proposed in this study.

## 3 Research design

This research employed a two-study design to examine how GAI advocacy–practice inconsistency is associated with art students’ cognitive appraisals and creative process engagement. The research was conducted in China from February to May 2026. Study 1 served as a preliminary examination of the appraisal component of the proposed model. Using a classroom-based between-subjects scenario experiment, it compared students’ challenge and hindrance appraisals under high- and low-inconsistency conditions, thereby providing initial evidence for the proposed relationships between GAI advocacy–practice inconsistency and the two appraisal responses. Study 2 extended this preliminary examination to students’ actual GAI-supported art-learning experiences and tested the full conceptual model, including the indirect relationships through challenge and hindrance appraisals and the moderating role of pathways thinking. Accordingly, the two studies progressed from an initial examination of the focal appraisal relationships to a more comprehensive assessment of the proposed model and its boundary conditions.

Different analytical strategies were employed in accordance with the purpose of each study. In Study 1, independent-samples t-tests were used to compare challenge and hindrance appraisals across the high- and low-inconsistency conditions. This approach was appropriate because Study 1 involved two independent experimental groups and tested whether the mean levels of the two continuous appraisal outcomes differed between conditions. In Study 2, partial least squares structural equation modeling (PLS-SEM) was conducted using SmartPLS 4.1. The analysis proceeded by first evaluating the measurement model in terms of indicator reliability, internal consistency, convergent validity, and discriminant validity, followed by assessment of the structural model, including collinearity, explained variance, predictive relevance, and the hypothesized direct, indirect, moderating, and conditional indirect relationships. Statistical significance was assessed using bootstrapping with 5,000 resamples. PLS-SEM was selected because the study focused on explaining variance in the endogenous constructs while simultaneously estimating a structurally complex model involving a higher-order construct, interaction terms, and multiple indirect relationships, making it well suited to the analytical objectives of the present study (Hair et al., 2019). Relative to a CB-SEM approach primarily oriented toward covariance reproduction and global model-fit assessment, PLS-SEM was therefore more closely aligned with the variance-oriented and structurally complex analytical objectives of this study. Table 1 summarizes the research design, analytical techniques, and research purposes of the two studies.

## 4 Study 1

### 4.1 Participants and procedure

Study 1 employed a classroom-based between-subjects scenario experiment with undergraduate students enrolled in art-related majors at two universities in China. Participants were recruited in February to March of 2026 through departmental instructors during regular classroom sessions. To be eligible, participants were required to be currently enrolled as undergraduate students in an art-related major and to provide informed consent before participation. The target sample was set at 100 participants per condition, yielding an initial sample of 200 students. Eligible participants were randomly assigned in approximately equal numbers to the high- and low-inconsistency conditions. Responses were screened according to prespecified data-quality criteria. Responses were excluded if participants failed either of the scenario-comprehension checks, failed the instructed-response attention check, or exhibited clear straight-line or patterned responding. A total of 40 responses were excluded during data screening, resulting in a final sample of 160 valid cases: 82 in the low-inconsistency condition and 78 in the high-inconsistency condition. Participants averaged 20.84 years of age (*SD* = 1.30), with women accounting for 51.2% of the sample (*n* = 82). Most participants reported at least moderate familiarity with GAI tools. The experimental session required approximately 10–15 min to complete.

The scenario manipulation was developed according to three design principles. First, both conditions shared the same overarching institutional background and the same stated commitments regarding the integration of GAI into multiple art courses. Second, the experimental contrast was constructed through a direct correspondence between these prior commitments and their subsequent implementation. Both scenarios covered the same substantive domains, including usage guidance, training and technical resources, instructional requirements, assessment standards, and responses to emerging problems, but differed in whether the announced commitments were subsequently enacted in practice. Third, implementation problems in the high-inconsistency condition were presented as direct discrepancies from prior commitments rather than as unrelated negative events, whereas the corresponding commitments in the low-inconsistency condition were broadly implemented as stated.

In the high-inconsistency condition, guidance remained unclear, promised training and resources were unevenly available, instructors applied conflicting requirements, assessment practices departed from previously stated standards, and reported problems remained unresolved. In the low-inconsistency condition, the corresponding commitments were broadly implemented through clear guidance, planned resource provision, coordinated instructional requirements, consistent assessment practices, and follow-up clarification of emerging issues. Thus, both scenarios retained the same overarching GAI-supported art-learning context and institutional advocacy, with the intended experimental contrast centered on the correspondence between stated commitments and subsequent practices. Participants were instructed to imagine studying in the described environment across multiple courses throughout one academic year and then evaluate it in terms of challenge and hindrance appraisals. The two scenario versions followed the same format and administration procedure.

### 4.2 Measurement

All measures used in Study 1 were based on previously validated scales and were adapted for the GAI-supported art-education context using a construct-preserving procedure. The number of items and the core conceptual content of the original measures were retained, while the referent and contextual wording were adjusted only where necessary. The original English items were translated into Chinese and then independently back-translated into English. The translated and back-translated versions were compared with the original scales, and any discrepancies were resolved with emphasis on semantic equivalence rather than literal wording. The contextualized items were subsequently reviewed by two researchers with expertise in educational psychology, semantic clarity, and contextual appropriateness. Minor wording refinements were made where necessary before the measures were finalized. Unless otherwise specified, all items were rated on a 7-point Likert scale ranging from 1 (strongly disagree) to 7 (strongly agree).

Challenge and hindrance appraisals were measured using the scales developed by LePine et al. (2016), with three items for each construct. The original scales assessed whether work demands were perceived as facilitating or constraining personal growth, goal attainment, and accomplishment. These appraisal criteria were retained, while the referent was changed to the ongoing GAI-supported art-learning environment described in the experimental scenario. Challenge appraisal captured the extent to which participants perceived the environment as facilitating growth, goal attainment, and accomplishment in art learning and creative work, whereas hindrance appraisal captured the extent to which the same environment was perceived as constraining these outcomes. The final wording of these six appraisal items was retained unchanged in Study 2 to maximize measurement comparability across the two studies.

The experimental manipulation was assessed using five items adapted from the organizational hypocrisy scale developed by Kılıçoğlu et al. (2019). The adaptation preserved the substantive forms of word–deed inconsistency represented in the original scale while locating them within institutional GAI education. The resulting items assessed discrepancies between the school’s stated GAI-related commitments and its subsequent practices concerning institutional support, implementation, established guidelines, assessment, and the handling of emerging problems. Accordingly, implementation-related difficulties were considered as manifestations of discrepancies between institutional commitments and practices rather than as independent aspects of implementation quality. The five items were averaged, with higher scores indicating greater perceived GAI advocacy–practice inconsistency. In Study 1, this measure was used solely as a manipulation check and was not treated as an outcome variable.

### 4.3 Manipulation check

An independent-samples t-test was conducted to determine whether perceived GAI advocacy–practice inconsistency differed significantly between the two experimental conditions. Participants in the high-inconsistency condition reported significantly higher perceived inconsistency (*M* = 4.78, *SD* = 1.01) than those in the low-inconsistency condition (*M* = 2.83, *SD* = 0.85), *t*(158) = 13.28, *p* < 0.001, Cohen’s *d* = 2.10. These results indicate that participants clearly distinguished the two conditions in terms of the intended perceived GAI advocacy–practice inconsistency manipulation. However, because implementation features such as guidance clarity, support availability, and assessment predictability were embedded within the scenarios, the manipulation should be interpreted as representing a broader experience of inconsistent implementation rather than a completely isolated effect of word–deed discrepancy.

### 4.4 Results

The study further examined whether the two experimental conditions differed in challenge and hindrance appraisals. Participants in the high-inconsistency condition reported significantly higher challenge appraisal (*M* = 4.58, *SD* = 1.30) than those in the low-inconsistency condition (*M* = 2.96, *SD* = 1.37), *t*(158) = 7.68, *p* < 0.001, Cohen’s *d* = 1.22. Participants in the high-inconsistency condition also reported significantly higher hindrance appraisal (*M* = 4.71, *SD* = 1.27) than those in the low-inconsistency condition (*M* = 3.30, *SD* = 1.38), *t*(158) = 6.71, *p* < 0.001, Cohen’s *d* = 1.06. Thus, participants assigned to the high-inconsistency scenario reported stronger challenge and hindrance appraisals than those assigned to the low-inconsistency scenario, providing scenario-based support for H1a and H2a.

## 5 Study 2

### 5.1 Participants and procedure

Study 2 employed a cross-sectional online survey to examine the full conceptual model using students’ actual experiences of GAI-supported art learning and creation. Data were collected between April and May 2026 through Wenjuanxing (Questionnaire Star), an online survey platform widely used in China. Participants were recruited from art-related programs at higher education institutions across China through departmental instructors, course-based student communication groups, and student associations or online communities related to art and design. To be eligible, respondents were required to be at least 18 years old, currently enrolled in an art-related program, and to have used GAI tools at least once during the preceding 6 months to support art learning or creative work.

A total of 546 questionnaires were returned. Responses were screened according to prespecified data-quality criteria. Questionnaires were excluded if respondents did not meet the eligibility requirements, failed the instructed-response attention check, submitted incomplete responses, completed the survey in less than 5 min, or exhibited clear straight-line or patterned responding. After excluding 121 responses, 425 questionnaires were retained for analysis. The participants’ mean age was 21.61 years (*SD* = 2.29). The final sample comprised 228 female (53.6%) and 197 male (46.4%). Regarding current level of study, 178 participants (41.9%) were enrolled in junior college programs, 182 (42.8%) in undergraduate programs, and 65 (15.3%) in postgraduate programs. Participants were drawn from comprehensive universities (27.3%), specialized arts institutions (33.9%), and other types of higher education institutions (38.8%). In addition, 77.4% reported at least a moderate level of familiarity with GAI tools.

### 5.2 Measurement

Study 2 used the finalized measures developed through the translation, contextual adaptation, and content-review procedures described in section 4.2. Unless otherwise specified, all focal constructs were measured using 7-point Likert scales ranging from 1 (strongly disagree) to 7 (strongly agree). Whereas Study 1 responses were anchored to the experimentally presented academic-year environment, Study 2 participants responded with reference to their own experiences of GAI-supported art learning and creation.

GAI advocacy–practice inconsistency was measured using the same five-item scale that served as the manipulation-check measure in Study 1. In Study 2, participants responded to these items based on their own experiences of GAI-supported art learning and their perceptions of the consistency between institutional commitments and practices. Higher scores indicated greater perceived inconsistency between the school’s stated GAI-related commitments and its subsequent implementation.

Pathways thinking was assessed using four items from Snyder et al. (1991). Because pathways thinking was conceptualized as a relatively general individual tendency rather than a GAI-specific situational response, its original substantive wording was retained rather than contextualized to GAI or art education. The items captured participants’ perceived capacity to generate multiple routes around difficulties and toward important goals.

Challenge and hindrance appraisals were each assessed using the same three-item scales used in Study 1 (LePine et al., 2016). The item wording was identical across the two studies; only the source of the focal experience differed. In Study 2, participants were instructed to evaluate the continuing GAI-supported learning environment on the basis of their school’s advocacy, implementation arrangements, instructor requirements, assessment practices, and their own actual educational experiences. This design maintained a common measurement referent while allowing Study 1 to capture scenario-based appraisals and Study 2 to capture experience-based appraisals.

Creative process engagement was assessed using the 11-item scale developed by Zhang and Bartol (2010). The original three-dimensional structure and item allocation were retained: problem identification (three items), information searching and encoding (three items), and idea generation (five items). The items were contextualized to participants’ most recent GAI-assisted art-learning or creation task while preserving the behavioral meaning of the original dimensions. Thus, the adapted items continued to assess cognitive activities such as understanding and decomposing the creative problem, searching for and retaining task-relevant information from multiple sources, and generating, comparing, and refining alternative ideas. In the PLS-SEM model, creative process engagement was specified as a reflective–reflective hierarchical component model, with individual items modeled as reflective indicators of their respective first-order dimensions and the three first-order dimensions modeled as reflective manifestations of the higher-order creative process engagement construct. Given the higher-order specification of creative process engagement, the disjoint two-stage approach was used for estimation (Sarstedt et al., 2019). In the first stage, the three lower-order components, problem identification, information searching and encoding, and idea generation, were estimated and evaluated for reliability and validity. In the second stage, their latent variable scores were used as reflective indicators of the higher-order creative process engagement construct.

**Table 1:** The research design of this study.

Study	Research design	Analytical technique	Research purpose
Study 1	Classroom-based between-subjects scenario experiment	Independent-samples *t*-tests	Provide an initial test of whether GAI advocacy–practice inconsistency evokes challenge and hindrance appraisals.
Study 2	Cross-sectional online survey	PLS-SEM with bootstrapping	Test the full model, including direct, indirect, moderating, and conditional indirect relationships.

A four-item attitude-toward-the-color-blue scale developed by Miller and Simmering (2023) was included as a theoretically unrelated marker variable for the assessment of common method bias (CMB). The marker construct was used only for diagnostic purposes and was not included as a substantive variable in the hypothesized model. Gender, current level of study, and familiarity with GAI tools were included as baseline control variables. These variables were selected to account for basic demographic, educational-stage, and technology-experience differences that could covary with students’ engagement in GAI-supported creative activities.

### 5.3 CMB test

Because Study 2 relied on cross-sectional self-report data collected through a single survey, Harman’s single-factor test was used as an initial diagnostic assessment of potential CMB, and the marker-variable technique was further employed as a complementary assessment. Harman’s single-factor test showed that the first unrotated factor explained 25.6% of the total variance, below the 50% threshold. Moreover, the marker variable (Table 2) was not significantly related to challenge appraisal (*β* = −0.073, *p* = 0.281), creative process engagement (*β* = −0.053, *p* = 0.445), or hindrance appraisal (*β* = 0.021, *p* = 0.722). The changes in *R*^2^ after including the marker variable ranged from 0.001 to 0.005. These diagnostic results did not indicate a severe common method bias problem in the present data (Lindell and Whitney, 2001; Podsakoff et al., 2003).

**Table 2:** CMB assessment using the marker-variable technique.

Path	β value	LLCI	ULCI	*p-*value	*R* ^2^	Original *R*^2^
MV → CA	−0.073	−0.157	0.109	0.281	0.294	0.289
MV → CPE	−0.053	−0.151	0.113	0.445	0.210	0.208
MV → HA	0.021	−0.096	0.127	0.722	0.190	0.189

MV, marker variable; CA, challenge appraisal; CPE, creative process engagement; HA, hindrance appraisal.

### 5.4 Evaluating the measurement model

Following the disjoint two-stage procedure described in section 5.2, the measurement properties of the lower-order components were evaluated first, followed by assessment of the higher-order creative process engagement construct. As shown in Table 3, all first-order indicator loadings ranged from 0.756 to 0.931. Cronbach’s α values ranged from 0.816 to 0.890, CR values from 0.891 to 0.925, and AVE values from 0.676 to 0.805, indicating satisfactory reliability and convergent validity. At the higher-order level, creative process engagement showed adequate reliability and convergent validity, with Cronbach’s α of 0.907, composite reliability of 0.877, and AVE of 0.703. Its three first-order dimensions loaded strongly on the higher-order construct, with loadings ranging from 0.821 to 0.850.

**Table 3:** Indicator loadings, construct reliability, and convergent validity.

Construct	First order	Cronbach’s α	CR	AVE	Loading
GAI advocacy-practice inconsistency (API)	API1	0.880	0.912	0.676	0.803
	API2				0.802
	API3				0.858
	API4				0.848
	API5				0.797
Challenge appraisal (CA)	CA1	0.816	0.891	0.731	0.867
	CA2				0.853
	CA3				0.845
Hindrance appraisal (HA)	HA1	0.829	0.897	0.744	0.854
	HA2				0.873
	HA3				0.861
Idea generation (IG)	IG1	0.890	0.919	0.695	0.781
	IG2				0.854
	IG3				0.856
	IG4				0.86
	IG5				0.813
Information searching and encoding (ISE)	ISE1	0.848	0.908	0.768	0.886
	ISE2				0.912
	ISE3				0.828
Problem identification (PI)	PI1	0.878	0.925	0.805	0.850
	PI2				0.909
	PI3				0.931
Pathways thinking (PT)	PT1	0.872	0.904	0.704	0.756
	PT2				0.799
	PT3				0.910
	PT4				0.881
Construct	Second order	Cronbach’s α	CR	AVE	Loading
Creative process engagement (CPE)	PI	0.907	0.877	0.703	0.85
	ISE				0.844
	IG				0.821

PI, problem identification; ISE, information searching and encoding; IG, idea generation. PI, ISE, and IG represent the three first-order components of the second-order CPE construct. CR, composite reliability; AVE, average variance extracted.

Following the robustness-check recommendations for PLS-SEM by Vaithilingam et al. (2024), skewness and kurtosis were examined to assess potential departures from univariate normality. As reported in Table 4, skewness values ranged from −0.067 to 0.252 and kurtosis values from −1.320 to −0.752, indicating no substantial univariate distributional problems. The square root of each construct’s AVE exceeded its correlations with other constructs, and all HTMT values were below 0.85, with a maximum of 0.805. These results support discriminant validity (Henseler et al., 2015).

**Table 4:** Descriptive statistics and discriminant validity.

Construct	Skewness	Kurtosis	1	2	3	4	5	6	7
1. API	0.064	−1.110	**0.822**	0.573	0.50	0.173	0.116	0.088	0.076
2. CA	0.117	−1.229	0.487	**0.855**	0.119	0.354	0.474	0.463	0.114
3. HA	−0.058	−1.160	0.431	0.096	**0.863**	0.111	0.245	0.209	0.045
4. CPE-IG	−0.067	−1.096	0.155	0.302	0.096	**0.833**	0.558	0.551	0.033
5. CPE-ISE	0.224	−0.752	0.090	0.401	−0.204	0.486	**0.876**	0.805	0.092
6. CPE-PI	0.252	−0.957	0.077	0.392	−0.178	0.488	0.699	**0.897**	0.061
7. PT	0.035	−1.320	−0.068	0.118	−0.040	−0.012	0.071	0.048	**0.839**

API, GAI advocacy-practice inconsistency, CA, challenge appraisal, HA, hindrance appraisal, PT, pathways thinking, CPE, creative process engagement. Diagonal = $\sqrt{\mathrm{AVE}\;}$ ; Below diagonal: estimated correlations; Above diagonal: HTMT ratios. The bold diagonal values represent the square roots of the AVE.

### 5.5 Hypothesis testing

The structural model was estimated using PLS-SEM in SmartPLS 4.1. Statistical significance was assessed through bootstrapping with 5,000 resamples. The inner VIF values ranged from 1.000 to 1.025, indicating no problematic collinearity. The model accounted for 18.9, 28.9, and 20.8% of the variance in the three endogenous constructs, with corresponding *Q*^2^ values of 0.133, 0.206, and 0.103, respectively, supporting its predictive relevance (Hair et al., 2019).

As shown in Table 5, GAI advocacy–practice inconsistency was positively associated with hindrance appraisal (*β* = 0.431, *p* < 0.001) and challenge appraisal (*β* = 0.496, *p* < 0.001), supporting H1a and H2a. The estimated indirect association between inconsistency and creative process engagement through hindrance appraisal was negative and significant (*β* = −0.060, *p* = 0.004), whereas the estimated indirect association through challenge appraisal was positive and significant (*β* = 0.217, *p* < 0.001), supporting H1b and H2b.

**Table 5:** Results of hypothesis testing.

Hypothesis	Structural relationship	β	LLCI	ULCI	*p-*value	VIF	Support
H1a	API → HA	0.431	0.347	0.514	<0.001	1.005	Yes
H1b	API → HA → CPE	−0.060	−0.103	−0.022	0.004	–	Yes
H2a	API → CA	0.496	0.420	0.573	<0.001	1.005	Yes
H2b	API → CA → CPE	0.217	0.168	0.270	<0.001	–	Yes
H3a	PT × API → HA	−0.060	−0.148	0.028	0.182	1.000	No
H3b	PT × API → CA	0.172	0.087	0.240	<0.001	1.000	Yes
H3c	PT × API → HA → CPE	0.008	−0.004	0.024	0.251	–	No
H3d	PT × API → CA → CPE	0.075	0.037	0.110	<0.001	–	Yes

API, GAI advocacy–practice inconsistency; HA, hindrance appraisal; CA, challenge appraisal; PT, pathways thinking; CPE, creative process engagement; LLCI, lower limit of the confidence interval; ULCI, upper limit of the confidence interval; VIF, variance inflation factor.

Pathways thinking did not significantly moderate the relationship between inconsistency and hindrance appraisal (*β* = −0.060, *p* = 0.182), and the corresponding conditional indirect association was nonsignificant (*β* = 0.008, *p* = 0.251). Thus, H3a and H3c were not supported. In contrast, the interaction between pathways thinking and inconsistency was positively associated with challenge appraisal (*β* = 0.172, *p* < 0.001), and the corresponding positive conditional indirect association through challenge appraisal was significant (*β* = 0.075, *p* < 0.001), supporting H3b and H3d. As shown in Table 6, no control variable significantly predicted creative process engagement.

**Table 6:** Effects of control variables.

Control relationship	β	LLCI	ULCI	*p* value	VIF
Gender → CPE	0.045	−0.130	0.217	0.612	1.025
Education level → CPE	−0.038	−0.125	0.046	0.383	1.010
GAI familiarity → CPE	−0.043	−0.121	0.035	0.278	1.011

CPE, creative process engagement; GAI, generative artificial intelligence; LLCI, lower limit of the confidence interval; ULCI, upper limit of the confidence interval; VIF, variance inflation factor.

## 6 Discussion

### 6.1 Discussion of results

This study examined how GAI advocacy–practice inconsistency relates to art students’ creative process engagement through cognitive appraisals, and whether the associations between inconsistency and these appraisals differ across levels of pathways thinking. Study 1 showed that students assigned to the high-inconsistency scenario reported stronger challenge and hindrance appraisals than those assigned to the low-inconsistency scenario. Given the bundled nature of the scenario manipulation, this finding should be interpreted as reflecting a broader experience of inconsistent implementation rather than an isolated word–deed inconsistency effect. Study 2 showed a corresponding pattern in students’ actual educational experiences, with perceived GAI advocacy–practice inconsistency positively associated with both challenge and hindrance appraisals. The two studies provide complementary evidence for the proposed dual-appraisal pattern. These findings are consistent with the transactional view that the same environmental condition may be appraised as both an obstacle to goal attainment and an opportunity for adaptation or growth (Lazarus and Folkman, 1984; Webster et al., 2011; Podsakoff et al., 2023).

The two appraisal pathways were associated with creative process engagement in opposite directions. A significant negative indirect association was observed between GAI advocacy–practice inconsistency and creative process engagement through hindrance appraisal. Students who reported stronger hindrance appraisal also tended to report lower engagement in core creative processes. By contrast, a significant positive indirect association was observed through challenge appraisal, with stronger challenge appraisal associated with greater cognitive investment in GAI-assisted creative activities. These findings align with evidence that hindrance appraisals tend to undermine engagement, while challenge appraisals foster greater effort and more proactive coping responses (LePine et al., 2016; Kronenwett and Rigotti, 2022; Liu et al., 2022). Importantly, these findings do not imply that institutional inconsistency is beneficial in itself; rather, they indicate that its consequences depend on how students evaluate its implications for goal attainment.

Pathways thinking strengthened the positive association between GAI advocacy–practice inconsistency and challenge appraisal, and the corresponding positive conditional indirect association with creative process engagement was also stronger at higher levels of pathways thinking. Individuals high in pathways thinking are better able to devise workable alternatives when an intended course of action becomes obstructed. Accordingly, they may interpret inconsistent institutional practices not merely as barriers, but as situations that can be navigated through experimentation, adaptation, and the pursuit of alternative solutions (Snyder et al., 1991; Gallagher et al., 2021). However, pathways thinking did not significantly alter the association between inconsistency and hindrance appraisal, nor the adverse indirect effect operating through hindrance appraisal. This finding suggests that pathways thinking does not necessarily reduce students’ perceptions of institutional constraints themselves. Although students with stronger pathways thinking may identify alternative courses of action and perceive greater possibilities for responding to inconsistent institutional practices, they may still recognize unclear expectations, insufficient resources, or inconsistent implementation as genuine barriers within the learning environment. Therefore, challenge and hindrance appraisals may coexist because students can simultaneously perceive environmental constraints and opportunities for action in response to the same institutional condition (Webster et al., 2011; Podsakoff et al., 2023). In this sense, the adaptive role of pathways thinking may lie primarily in expanding perceived action possibilities rather than reducing perceived stress associated with institutional inconsistency. Rather than eliminating negative evaluations of the environment, pathways thinking helps students navigate demanding situations by increasing their available response options, which explains why it strengthened the challenge pathway but did not significantly weaken the hindrance pathway.

### 6.2 Theoretical implications

This study advances understanding of GAI-supported art education by highlighting how institutional inconsistency, cognitive appraisal processes, and individual cognitive resources jointly shape students’ creative engagement. The theoretical contributions are as follows:

First, this study introduces GAI advocacy–practice inconsistency as an important institutional condition in GAI-supported art education. Drawing on the concept of organizational word–deed alignment (Simons, 2002; Kılıçoğlu et al., 2019), this study conceptualizes GAI advocacy–practice inconsistency as a discrepancy between institutional commitments regarding GAI integration and their actual implementation. By extending the word–deed alignment perspective to GAI-supported art learning, this study highlights that students’ experiences with educational AI are shaped not only by the availability of AI-related resources but also by whether institutional advocacy is consistently enacted in practice.

Second, this study extends the transactional perspective of stress and coping by demonstrating that institutional inconsistency can simultaneously evoke challenge and hindrance appraisals. Grounded in the transactional theory of stress and coping (Lazarus and Folkman, 1984) and challenge–hindrance appraisal research (Webster et al., 2011; Podsakoff et al., 2023), this study shows that GAI advocacy–practice inconsistency does not carry a fixed psychological meaning. Instead, the same institutional condition may be interpreted as both a constraint that interferes with goal attainment and a demand that stimulates adaptation and problem solving. This finding advances understanding of students’ responses to uncertain institutional changes by demonstrating that inconsistent educational environments may trigger dual appraisal processes rather than a single positive or negative response.

Finally, this study refines understanding of the role of individual cognitive resources within the appraisal process by suggesting an asymmetric pattern in the moderating role of pathways thinking. Consistent with the view that personal resources influence how individuals evaluate and cope with demanding situations (Hobfoll, 2001; Xanthopoulou et al., 2007), the findings indicate that pathways thinking strengthens challenge appraisal by helping students identify possible actions and adaptive responses under inconsistent institutional conditions. By contrast, no significant buffering effect was detected for hindrance appraisal or for the corresponding negative indirect pathway. Given that the buffering predictions for the hindrance pathway were theoretically tentative and supported by limited prior evidence, these nonsignificant findings should not be interpreted as demonstrating the absence of such an effect. Rather, they suggest that pathways thinking may be more effective in expanding perceived possibilities for action than in reducing perceptions of environmental obstruction. This pattern extends the TTSC perspective by suggesting that individual cognitive resources may facilitate more proactive interpretations of demanding situations without necessarily eliminating perceptions of environmental barriers.

### 6.3 Practical implications

The findings underscore the importance of aligning institutional advocacy with actual implementation. Universities and art schools should ensure that public commitments to GAI integration are supported by clear usage policies, adequate technical resources, consistent assessment standards, and effective mechanisms for resolving emerging problems. Such alignment is particularly important because inconsistent practices may encourage students to interpret the learning environment as obstructive, thereby weakening their engagement in creative activities. Institutional efforts should therefore focus not only on promoting GAI use but also on ensuring that policies are translated into coherent and predictable educational practices.

This institutional consistency should be reinforced at the course level. Instructors need to clarify how GAI may be used in specific assignments, what forms of assistance are acceptable, and how originality, revision, and process documentation will be evaluated. Clear expectations can reduce uncertainty and limit hindrance appraisal. At the same time, instructors can foster challenge appraisal by designing tasks that require students to compare alternative outputs, refine prompts, evaluate weaknesses in generated content, and justify their creative decisions. In this way, the complexity of GAI-assisted creation can be framed as a manageable learning demand rather than an unpredictable constraint.

Beyond reducing inconsistency, universities should also help students develop the capacity to identify alternative routes when difficulties arise. Activities such as tool comparison, contingency planning, and multiple-solution exploration may strengthen pathways thinking and support more constructive appraisals of uncertain learning conditions. However, the nonsignificant buffering effect on the hindrance pathway indicates that student adaptability cannot substitute for institutional responsibility. Accordingly, support for pathways thinking should complement, rather than replace, improvements in policy implementation and instructional coordination.

### 6.4 Limitations and future directions

The study has several limitations that warrant attention. First, Study 1 has limitations regarding the specificity and validation of the experimental manipulation. Although the scenarios were structured around whether the school’s stated GAI commitments were subsequently enacted in practice, the resulting inconsistency was conveyed through concrete implementation features, such as uneven support, conflicting instructional requirements, unpredictable assessment, and unresolved problems. Because these features were not manipulated independently, the study cannot fully distinguish the effect of advocacy–practice inconsistency from the specific implementation conditions through which that inconsistency was experienced. In addition, although scenario-comprehension checks were incorporated into the main experiment, the scenario materials were not independently pre-tested for perceived realism and comprehensibility before formal data collection. Emotional load and perceived confusion were also not separately assessed across the high- and low-inconsistency conditions, and their potential influence on participants’ appraisal responses therefore cannot be completely ruled out. Future research could conduct dedicated pre-tests of scenario realism, comprehensibility, emotional load, and perceived confusion, while also employing factorial designs that independently manipulate advocacy–practice alignment and particular implementation conditions, such as support adequacy or rule predictability, to examine their distinct and interactive effects on students’ appraisals.

Second, Study 2 relied on cross-sectional self-report data. Although combining a scenario-based experiment with evidence from students’ actual experiences provided complementary insights, the cross-sectional design does not establish temporal or causal ordering among GAI advocacy–practice inconsistency, cognitive appraisals, and creative process engagement. The estimated indirect and conditional indirect associations should therefore be interpreted as statistically consistent with the proposed theoretical model rather than as evidence of causal mediation. Reverse or reciprocal relationships also cannot be ruled out; for example, students who are more deeply engaged in GAI-assisted creative activities may subsequently evaluate the learning environment more positively as a challenge or become less sensitive to some hindering features. In addition, the predictors and creative process engagement were assessed at somewhat different referent scopes. GAI advocacy–practice inconsistency and the appraisal measures referred to the broader institutional GAI-supported learning environment, whereas creative process engagement referred to participants’ most recent GAI-assisted learning or creative task. Although this design linked broader contextual perceptions to engagement in a concrete recent episode, the difference in referent scope may introduce some measurement imprecision. Future research could use multi-wave, time-lagged, or experience-sampling designs to examine temporal ordering and align the focal constructs within the same task, course, or time period. Behavioral indicators, process records, or instructor evaluations could also complement students’ self-reports.

Third, the contextual scope and explanatory coverage of the model remain limited. Participants were recruited from art-related programs in China, and the findings may not generalize directly to other academic disciplines, educational systems, or sociocultural contexts. In addition, the model explained more variance in challenge and hindrance appraisals than in creative process engagement, suggesting that engagement is also shaped by factors beyond the present framework. Future research could test the model across more diverse student populations and institutional contexts and incorporate additional influences, such as task characteristics, instructional arrangements, students’ confidence in using GAI, and the availability of technical assistance.

## Data Availability

The raw data supporting the conclusions of this article will be made available by the authors, without undue reservation.
