# Supplementary material for: When universities advocate GAI but practice falls short: student appraisals and creative process engagement in art education
Source: Front Psychol. 2026 Sep 25;17:1955192. doi: 10.3389/fpsyg.2026.1955192 (PMC13649748; doi:10.3389/fpsyg.2026.1955192)
Supplement: Supplementary file 1 [file Supplementary_file_1.docx]

Supplementary Material

This supplementary material provides the experimental scenarios used in Study 1 and the measurement items used in Study 1 and Study 2. The materials correspond to those included in the final questionnaire administered to participants.

# 1 Supplementary Material S1. Study 1 Scenario Experiment Materials

### 共同背景（A、B两组相同） / Common Background (Identical in Both Conditions)

你是某大学艺术学院的一名本科生。近年来，生成式人工智能逐渐被用于绘画、视觉设计、数字媒体、影视、动画、音乐及其他艺术领域。为了推动艺术教育改革，你所在学院宣布，将在未来一个学年中把GAI逐步纳入本科艺术教学。

根据学院发布的方案，学生将在多门专业课程中接触GAI，包括创意构思、资料搜索、方案比较、作品修改和创作反思。学院同时要求学生记录GAI的使用过程，解释自己如何判断、选择和修改AI生成的内容，并保证最终作品体现学生本人的创作决策。

在接下来的一个学年中，你需要在多门课程中完成不同类型的艺术学习和创作任务。学院有关GAI的规定、教师的实际要求以及相关支持，会持续影响你的学习安排和创作过程。

请继续阅读学院在这一学年中的具体做法，并设想自己长期处于这样的学习环境中。

You are an undergraduate student at a university school of art. In recent years, generative artificial intelligence has increasingly been used in painting, visual design, digital media, film, animation, music, and other artistic fields. To promote educational innovation, your school announces that GAI will gradually be integrated into undergraduate art education over the coming academic year.

According to the school’s initiative, students will encounter GAI in several professional courses, including idea development, information searching, comparison of alternatives, revision of creative work, and reflection on the creative process. Students will also be required to document how they use GAI, explain how they evaluate, select, and revise AI-generated content, and ensure that the final work reflects their own creative decisions.

During the coming academic year, you will complete different art-learning and creation tasks across several courses. The school’s GAI policies, instructors’ actual requirements, and the support provided will continuously affect your learning arrangements and creative process.

Please continue reading about the school’s practices during this academic year and imagine that you are studying in this environment over time.

### 版本A：高GAI倡导—实践不一致 / Version A: High GAI Advocacy–Practice Inconsistency

在学年开始时，学院通过官方网站、教学会议和学生说明会强调，GAI是艺术教育创新的重要工具。学院表示，将帮助学生合理使用GAI拓展创作思路、比较不同方案、提高创作效率并反思自己的创作过程。学院还承诺为不同艺术专业制定清晰、可行的使用规范，为教师和学生提供持续培训、技术资源及统一的评价标准。

然而，在接下来一个学年的实际学习中，你逐渐发现，学院的实际做法与上述倡导并不一致。

学院提出学生应在多门课程中掌握GAI辅助创作能力，但没有为不同专业设置明确的学习步骤，部分要求超出了学生现有的技术基础和课程时间。学生需要在多个创作任务中使用或说明GAI，但学院很少提供具体示例，学生难以判断什么属于合理使用。

学院曾表示会提供持续培训、软件账号和技术支持，但相关安排并不稳定。有些课程可以使用所需工具，有些课程则无法获得相应资源。不同教师对GAI的态度和要求也不一致：有的教师鼓励使用，有的教师限制使用，还有的教师在课程开始时允许使用，但在评分时又对使用GAI的作品持否定态度。

学院公开要求学生如实记录GAI的使用过程，并表示透明披露不会使学生受到不利评价。但在实际课程中，即使学生按照要求完整记录，也可能被认为缺乏原创性或独立思考，并因此影响成绩。学生很难预先判断同一种GAI使用方式在不同课程中会得到怎样的评价。

在这一学年中，学生多次向学院反映，学院的公开倡导、课程要求和实际评价之间存在矛盾。学院通常表示会进一步协调和解决，但之后很少发布明确的处理结果，原有问题仍然反复出现。一些争议经过短暂讨论后便被搁置。

因此，在多门课程和多个创作任务中，你需要持续面对不断变化、彼此矛盾且难以预测的GAI相关要求，并自行寻找完成学习和创作任务的办法。请设想自己在整个学年中持续处于这种学习环境。

At the beginning of the academic year, the school emphasizes through its official website, teaching meetings, and student briefings that GAI is an important tool for innovation in art education. The school states that it will help students use GAI appropriately to expand creative ideas, compare alternatives, improve efficiency, and reflect on their creative process. It also promises clear and workable guidelines for different art disciplines, continuous training, technical resources, and consistent assessment standards.

However, over the following academic year, you gradually find that the school’s actual practices are inconsistent with this advocacy.

The school expects students to develop GAI-assisted creation skills across several courses, but it does not provide clear learning steps for different disciplines, and some requirements exceed students’ current technical foundation and available course time. Students are required to use or explain GAI in multiple assignments, but few concrete examples are provided, making it difficult to judge what counts as appropriate use.

The school stated that it would provide continuous training, software accounts, and technical support, but these arrangements are unstable. Some courses provide access to the necessary tools, while others do not. Instructors also differ in their attitudes and requirements: some encourage GAI use, some restrict it, and some initially allow it but later evaluate GAI-assisted work negatively.

The school publicly requires students to document their use of GAI and states that transparent disclosure will not disadvantage them. In practice, however, students who fully document their use may still be judged as lacking originality or independent thinking and may receive lower grades. Students cannot reliably predict how the same use of GAI will be evaluated across different courses.

During the academic year, students repeatedly report conflicts among the school’s public advocacy, course requirements, and actual assessment. The school usually says that it will coordinate and resolve the issues, but rarely provides clear outcomes, and the same problems continue to occur. Some disputes are briefly discussed and then set aside.

Thus, across several courses and creative assignments, you must continually deal with changing, contradictory, and unpredictable GAI-related requirements and find your own ways to complete your learning and creative work. Imagine that you remain in this learning environment throughout the academic year.

### 版本B：低GAI倡导—实践不一致 / Version B: Low GAI Advocacy–Practice Inconsistency

在学年开始时，学院通过官方网站、教学会议和学生说明会强调，GAI是艺术教育创新的重要工具。学院表示，将帮助学生合理使用GAI拓展创作思路、比较不同方案、提高创作效率并反思自己的创作过程。学院还承诺为不同艺术专业制定清晰、可行的使用规范，为教师和学生提供持续培训、技术资源及统一的评价标准。

在接下来一个学年的实际学习中，你逐渐发现，学院的实际做法与上述倡导基本一致。

学院提出学生应在多门课程中掌握GAI辅助创作能力，并根据不同专业设置了相应的学习步骤。课程要求具有一定难度，学生需要投入时间学习新的工具、记录使用过程并解释自己的创作判断，但学院提供了具体示例，使学生能够判断什么属于合理使用。

学院按照计划提供培训、软件账号和技术支持。虽然不同课程使用GAI的具体方式有所不同，但教师依据统一的基本规范说明课程要求。课程开始时公布的使用规则与最终评分标准保持一致，学生可以预先判断不同使用方式可能产生的评价结果。

学院要求学生如实记录GAI的使用过程，并按照公开说明，在评价中关注学生如何判断、选择和修改AI生成内容。学生不会仅仅因为使用GAI而受到不利评价，但仍需要证明自己的独立思考、专业技能和创作贡献。

在这一学年中，学生也会对某些GAI使用范围和评价标准提出疑问。学院会汇总这些问题，发布补充说明，并协调不同课程的执行方式。经过处理后，调整结果会向学生反馈，类似问题通常能够得到进一步澄清。

因此，在多门课程和多个创作任务中，你需要持续学习新的GAI工具，应对具有一定难度的创作要求，并寻找适合自己的学习和创作方式，但学院的公开倡导、实际安排和评价标准总体保持一致。请设想自己在整个学年中持续处于这种学习环境。

At the beginning of the academic year, the school emphasizes through its official website, teaching meetings, and student briefings that GAI is an important tool for innovation in art education. The school states that it will help students use GAI appropriately to expand creative ideas, compare alternatives, improve efficiency, and reflect on their creative process. It also promises clear and workable guidelines for different art disciplines, continuous training, technical resources, and consistent assessment standards.

Over the following academic year, you gradually find that the school’s actual practices are generally consistent with this advocacy.

The school expects students to develop GAI-assisted creation skills across several courses and provides learning steps suited to different disciplines. The requirements are challenging: students must invest time in learning new tools, documenting their use, and explaining their creative judgments. However, concrete examples are provided, allowing students to judge what counts as appropriate use.

The school provides training, software accounts, and technical support as planned. Although the specific use of GAI varies across courses, instructors explain their requirements using consistent basic guidelines. Rules announced at the beginning of each course remain consistent with final assessment standards, allowing students to anticipate how different forms of GAI use will be evaluated.

The school requires students to document their use of GAI and, as publicly stated, evaluates how students judge, select, and revise AI-generated content. Students are not disadvantaged merely for using GAI, although they must still demonstrate independent thinking, professional skills, and their own creative contribution.

During the academic year, students also raise questions about the permitted scope of GAI use and assessment standards. The school collects these questions, issues supplementary guidance, and coordinates implementation across courses. After adjustments are made, the outcomes are communicated to students, and similar issues are usually clarified.

Thus, across several courses and creative assignments, you must continually learn new GAI tools, respond to demanding creative requirements, and find suitable ways of learning and creating, while the school’s public advocacy, actual arrangements, and assessment standards remain generally consistent. Imagine that you remain in this learning environment throughout the academic year.

正式打印提示：每份受试者问卷只保留共同背景和A、B其中一个版本；不得让同一受试者同时看到两个版本。

Printing note: Each participant questionnaire must contain the common background and only one of Versions A or B. Never show both versions to the same participant.

# 2 Supplementary Material S2. Study 1 Measurement Items

**Challenge Appraisal**

| Item | Statement |
| --- | --- |
| CA1 | 这一环境有助于促进我在艺术学习和创作中的成长与发展。  This environment helps promote my growth and development in art learning and creative work. |
| CA2 | 这一环境会激励我努力实现自己的学习和创作目标。  This environment motivates me to work toward my learning and creative goals. |
| CA3 | 总体而言，这一环境有助于促进我在艺术学习和创作中取得成就。  Overall, this environment helps promote my accomplishment in art learning and creative work. |

**Hindrance Appraisal**

| Item | Statement |
| --- | --- |
| HA1 | 这一环境会阻碍我在艺术学习和创作中的成长与发展。  This environment hinders my growth and development in art learning and creative work. |
| HA2 | 这一环境会限制我实现学习和创作目标的机会。  This environment limits my opportunities to achieve my learning and creative goals. |
| HA3 | 总体而言，这一环境会妨碍我在艺术学习和创作中取得成就。  Overall, this environment hinders my accomplishment in art learning and creative work. |

**GAI Advocacy–Practice Inconsistency (Manipulation Check)**

| Item | Statement |
| --- | --- |
| API1 | *学院管理层虽然表示会解决GAI教育或使用中出现的问题，但实际上这些问题并没有得到解决。*  *Although school management stated that it would address problems arising from GAI education or use, these problems remained unresolved in practice.* |
| API2 | *学院在GAI教育或使用方面提出的目标，与其实际能够提供的条件和支持并不相符。*  *The goals set by the school regarding GAI education or use were inconsistent with the conditions and support it actually provided.* |
| API3 | *学院在GAI教育或使用方面的实际做法，与其自行制定的规范或原则相违背。*  *The school’s actual practices regarding GAI education or use were contrary to the guidelines or principles it had established.* |
| API4 | *即使学院管理层公开表示要以某种方式推进GAI艺术教育，实际执行时却采取了不同的做法。*  *Even when school management publicly stated that it would implement GAI-based art education in a particular way, it did something different in practice.* |
| API5 | *学院在GAI教育或使用方面出现的争议或问题，被迅速搁置或掩盖，而没有得到公开、明确的处理。*  *Disputes or problems concerning GAI education or use in the school were quickly set aside or covered up rather than being addressed openly and clearly.* |

# 3 Supplementary Material S3. Study 2 Measurement Items

## **GAI Advocacy–Practice Inconsistency**

| Item | Statement |
| --- | --- |
| API1 | *学院管理层虽然表示会解决GAI教育或使用中出现的问题，但实际上这些问题并没有得到解决。*  *Although school management stated that it would address problems arising from GAI education or use, these problems remained unresolved in practice.* |
| API2 | *学院在GAI教育或使用方面提出的目标，与其实际能够提供的条件和支持并不相符。*  *The goals set by the school regarding GAI education or use were inconsistent with the conditions and support it actually provided.* |
| API3 | *学院在GAI教育或使用方面的实际做法，与其自行制定的规范或原则相违背。*  *The school’s actual practices regarding GAI education or use were contrary to the guidelines or principles it had established.* |
| API4 | *即使学院管理层公开表示要以某种方式推进GAI艺术教育，实际执行时却采取了不同的做法。*  *Even when school management publicly stated that it would implement GAI-based art education in a particular way, it did something different in practice.* |
| API5 | *学院在GAI教育或使用方面出现的争议或问题，被迅速搁置或掩盖，而没有得到公开、明确的处理。*  *Disputes or problems concerning GAI education or use in the school were quickly set aside or covered up rather than being addressed openly and clearly.* |

## **Pathways Thinking**

| Item | Statement |
| --- | --- |
| PT1 | 当我陷入困境时，我能够想到许多摆脱困境的办法。  I can think of many ways to get out of a jam. |
| PT2 | 对于任何问题，我通常都能找到多种解决途径。  There are lots of ways around any problem. |
| PT3 | 对于生活中对我最重要的事情，我能够想到多种实现方式。  I can think of many ways to get the things in life that are most important to me. |
| PT4 | 即使其他人感到泄气，我仍相信自己能够找到解决问题的办法。  Even when others get discouraged, I know I can find a way to solve the problem. |

## **Challenge Appraisal**

| Item | Statement |
| --- | --- |
| CA1 | 这一环境有助于促进我在艺术学习和创作中的成长与发展。  This environment helps promote my growth and development in art learning and creative work. |
| CA2 | 这一环境会激励我努力实现自己的学习和创作目标。  This environment motivates me to work toward my learning and creative goals. |
| CA3 | 总体而言，这一环境有助于促进我在艺术学习和创作中取得成就。  Overall, this environment helps promote my accomplishment in art learning and creative work. |

## **Hindrance Appraisal**

| Item | Statement |
| --- | --- |
| HA1 | 这一环境会阻碍我在艺术学习和创作中的成长与发展。  This environment hinders my growth and development in art learning and creative work. |
| HA2 | 这一环境会限制我实现学习和创作目标的机会。  This environment limits my opportunities to achieve my learning and creative goals. |
| HA3 | 总体而言，这一环境会妨碍我在艺术学习和创作中取得成就。  Overall, this environment hinders my accomplishment in art learning and creative work. |

## **Marker Variable: Attitude Toward the Color Blue**

| Item | Statement |
| --- | --- |
| MV1 | 蓝色是一种可爱的颜色。  Blue is a lovely color. |
| MV2 | 蓝色是一种令人愉快的颜色。  Blue is a pleasant color. |
| MV3 | 蓝色是一种不错的颜色。  Blue is a nice color. |
| MV4 | 我认为蓝色是一种漂亮的颜色。  I think blue is a pretty color. |

## **Creative Process Engagement**

| Item | Statement |
| --- | --- |
| PI1 | 我会花费较多时间理解艺术创作问题或任务的核心要求。  I spend considerable time trying to understand the core requirements of the art-creation problem or assignment. |
| PI2 | 我会结合自己的判断和GAI提供的内容，从多个角度思考创作问题。  I think about the creative problem from multiple perspectives by combining my own judgment with content provided by GAI. |
| PI3 | 我会将困难的艺术创作问题或任务分解成若干部分，以便进一步判断GAI可以在哪些环节提供帮助。  I break a difficult art-creation problem or assignment into parts to better determine where GAI may be helpful. |
| ISE1 | 我会查阅与艺术创作任务有关的多种信息，包括GAI生成的信息和其他资料。  I consult a wide variety of information relevant to the art-creation task, including GAI-generated information and other materials. |
| ISE2 | 我会从多个来源搜索信息，例如个人经验、他人作品、专业资料、互联网和GAI工具。  I search for information from multiple sources, such as personal experience, others’ work, professional materials, the Internet, and GAI tools. |
| ISE3 | 我会保留与GAI辅助艺术学习或创作有关的详细信息，以供今后的创作任务使用。  I retain detailed information related to GAI-assisted art learning or creation for future creative tasks. |
| IG1 | 在产生新的艺术创意时，我会综合考虑来自GAI和其他来源的信息。  When generating new artistic ideas, I consider information from GAI and other sources. |
| IG2 | 我会借助GAI或其他资料，寻找当前创作问题与不同艺术领域或解决方案之间的联系。  I use GAI or other materials to look for connections between the current creative problem and solutions from different artistic fields. |
| IG3 | 在确定最终创作方案之前，我会借助GAI或其他方式形成多个备选方案。  Before choosing a final creative solution, I generate multiple alternatives with the help of GAI or other approaches. |
| IG4 | 我会尝试结合GAI提供的内容，形成不同于既有做法的创作方案。  I try to combine content provided by GAI to develop creative solutions that depart from established approaches. |
| IG5 | 我会花费较多时间筛选和判断GAI及其他来源提供的信息，以形成新的艺术创意。  I spend considerable time evaluating and selecting information from GAI and other sources to generate new artistic ideas. |
